# Investigation of PRRS Virus Infection in Hungarian Wild Boar Populations during Its Eradication from Domestic Pig Herds

**DOI:** 10.3390/ani14111537

**Published:** 2024-05-23

**Authors:** Ádám Bálint, Sándor Csányi, Imre Nemes, Hanna Bijl, István Szabó

**Affiliations:** 1 Veterinary Diagnostic Directorate, National Food Chain Safety Office, H-1143 Budapest, Hungary; balintad@nebih.gov.hu; 2 Department of Wildlife Biology and Management, Hungarian University of Agriculture and Life Sciences, H-2100 Gödöllő, Hungary; s.csanyi@gmail.com; 3 National Food Chain Safety Office, H-1024 Budapest, Hungary; nemesi@nebih.gov.hu; 4 National PRRS Eradication Committee, H-1021 Budapest, Hungary

**Keywords:** Porcine Reproductive and Respiratory Syndrome (PRRS), disease transmission, wild boar, domestic pig, *Sus scrofa*, seroprevalence, Hungary, wildlife management

## Abstract

**Simple Summary:**

Simple Summary: Porcine Reproductive and Respiratory Syndrome (PRRS) is a common disease in Suidae species that causes significant economic losses in the pig industry. This study investigates PRRS in Hungarian wild boar populations and the efforts for eradication of the virus from domestic pig herds. Given the economic significance of PRRS, understanding its prevalence in wild hosts like wild boar is crucial. We examine whether wild boars in Hungary carry the PRRS virus, the seroconversion rate, and the potential relationship between wild boar and domestic pig populations concerning PRRS infection. Our findings indicate a minimal PRRS infection in wild boars, emphasizing the low risk they pose to the PRRS-free status of domestic pig herds. This research contributes to our understanding of PRRS dynamics in wild populations and supports efforts to maintain disease-free domestic pig herds, ensuring the sustainability of pig farming in Hungary.

**Abstract:**

Porcine Reproductive and Respiratory Syndrome (PRRS) significantly impacts the pig farming industry globally, leading to economic losses due to reduced productivity. This study focuses on assessing the presence and impact of PRRS within Hungarian wild boar populations amidst efforts to eradicate the virus from domestic pig herds. We used a combination of serological and virological tests on samples collected from wild boars across Hungary to evaluate the prevalence of PRRS virus and its potential transmission risks to domestic pigs. Our findings reveal a low seropositivity rate in wild boars, suggesting a minimal role of wild boars in the transmission of PRRS to domestic pig populations. Moreover, no relationship was found between domestic pig and wild boar densities, emphasizing the limited interaction and consequent risk of disease spread between these populations. We confirm the effectiveness of Hungary’s PRRS eradication measures among domestic herds and highlight the negligible risk posed by wild boars in re-introducing the PRRS virus.

## 1. Introduction

Porcine Reproductive and Respiratory Syndrome (PRRS) is recognized as one of the most economically significant infectious diseases affecting the pig industry worldwide [1]. It is estimated that an average PRRS outbreak reduces production by 7.4% and results in 1.92 fewer piglets per sow compared to disease-free intervals [2]. Financially, PRRS incurs substantial losses, with estimates in the United States pointing to costs of USD 114.71 per sow and USD 4.67 per slaughter pig [3], while in Scotland, the financial impact is calculated at GBP 80 per sow and GBP 3.5 per slaughter pig [4]. According to the CEO of Genesus, a leading genetics company, PRRS represents a greater loss to the pig industry than African Swine Fever (ASF) on a global scale [5]. Furthermore, a 2021 evaluation by Renken et al. [6], based on an analysis of 21 farms in Germany, projected the annual loss attributed to PRRS at EUR 255 per sow. In Hungary, the economic impact of PRRS infection on pig populations is approximated at HUF 5 billion (≈EUR 14 million) annually, affecting 170,000 breeding sows and approximately 4 million pigs until the end of the exemption period [7]. Susceptibility to the disease extends to various Suidae family members, including the common warthog (*Phacochoerus africanus*), bearded pig (*Sus barbatus*), and Cebu warty pig (*Sus cebifrons cebifrons*), yet natural vulnerability is predominantly observed in wild boars (*Sus scrofa*) and domestic pigs (*Sus scrofa domesticus*).

Pig-to-pig transmission (either indirectly or directly) is the most important factor in the spread of the disease pathogen. Direct transmission is dominated by intra-herd transmission, but also by trade in infected prefatteners [7]. Indirect sources of infection include activities related to deceased animal handling, live animal transport, semen distribution, and infected pig herds in closed units. This highlights the role of humans as vectors via mucous membranes and clothing. The virus’s viability extends to 11 days in the pig’s environment and 7 days in slurry [8,9]. The spread of the disease and infection are facilitated by large herds and areas with high pig densities.

PRRS is identified across the American and European continents, manifesting in two distinct genetic/antigenic varieties: the American and European types [10], with both showing various genetic divergences over recent decades. A 2014 survey revealed an average prevalence of PRRS in 71% of sows and 68% of weaned or fattening pigs, as reported by European experts [11].

The seropositivity and antigenicity of PRRS virus (PRRSV) have been confirmed in wild boar populations in Germany [12], France [13], the United States of America [14,15], Lithuania [16], Spain [17], Slovakia [18], China [19], Korea [20], and Japan [21]. The possibility of transmission between wild boar and domestic pigs exists, with wild boar potentially acting as reservoirs for infectious diseases affecting domestic pigs [22]. The potential for virus transmission between wild boar and domestic pigs has been extensively studied in relation to Aujeszky’s disease and ASF [13]. The outlined modes of transmission and infection between domestic pig herds could potentially affect feral pigs and vice versa, although the prevalence and distribution of PRRSV in wild boars remain uncertain [12]. Consequently, knowledge of PRRSV infection in wild boar populations may be of particular importance in areas aiming to eliminate PRRS from domestic herds [23].

In Hungary, PRRS virus was eradicated from domestic pig units between 2014 and 2022 (after the end of the eradication of Aujeszky’s disease [24]). The period between maintaining Aujeszky’s disease-free status and the PRRS eradication process highlighted concerns over wild boars being disease mediators in domestic pig units, posing re-infection risks. To completely exclude such direct or indirect contact, adhering to comprehensive measures is essential, including maintaining double fencing, using separate working attire, implementing hand and foot disinfection, eliminating green feeding, restricting access to hunters, outsiders, and vehicles, keeping litter and fodder away from wild boar, and adhering to waiting times.

The detection of the ASF virus in Heves County on 21 April 2018 caused a significant change in the number of wild boars in Hungary. The presence of ASF was confirmed only in wild boar, with no outbreaks in domestic pigs. In response, the National Chief Veterinarian issued an eradication plan for ASF in Hungary by Decision No 2/2021 [25]. This included a priority measure of reduction in the wild boar population by requiring all hunting concession holders to devise and submit a long-term, multi-annual action plan to the hunting authority for approval on the measures to be taken to achieve a maximum density of 0.5 wild boar per km^2^ (0.5 wild boar per 100 ha) in the wild by 28 February 2025.

In this study, we investigated whether the Hungarian wild boar populations are infected with PRRS virus, their seroconversion rates, and whether PRRS virus is detectable in wild boars in Hungary. Moreover, we assessed to what extent the population densities of wild boar and domestic pigs coincide, and whether any relationship can be detected between PRRS infection in domestic pig units and the spatial distribution of the wild boar population in Hungary. Our objective is to demonstrate the short- and long-term risks that the possible PRRS infection in wild boar populations poses to the declared PRRS-free status of domestic pig herds in 2022.

## 2. Materials and Methods

### 2.1. Data Collection

We collected data on the number of wild boars from Hungary’s National Game Management Database [26] and domestic pig populations from the Hungarian Central Statistical Office [27] in Hungary from 2011 to 2023. Using these data, we calculated the density of wild boars and domestic pigs per county and compared their spatial distribution. Furthermore, regular laboratory tests were carried out in connection with the PRRS clearance of herds of domestic pigs, the process and results of which were previously reported [23].

Blood samples were taken from live wild boars extensively kept in game gardens and game parks based on the National PRRS Eradication Programme. Samples were also taken within the framework of the official ASF monitoring programme from deceased as well as hunted wild boars in their natural habitat. We did not require approval from the bioethics committee, as this study did not involve experimental animals.

### 2.2. Laboratory Processing

The blood samples were tested for PRRS virus using ELISA and PCR. For PRRS ELISA, Ingezim PRRS 2.0 and IXEXX PRRS X3 Ab tests were used. Both tests detect IgG antibodies but cannot discriminate between the PRRS1 and PRRS2 types. The samples were inactivated in lysis buffer in BSL3 containment, and nucleic acid extraction and PCR were carried out in BSL2 conditions. The original samples were stored at −80 °C in BSL3 containment, whereas the extracted RNA was stored in a BSS2 laboratory also at −80 °C. Nucleic extraction was carried out with the MagAttract 96 cador Pathogen Kit (Qiagen, Hilden, Germany) using the KingFisher Flex system (Thermo Fisher, Waltham, MA, USA). Seropositivity was confirmed or ruled out by a secondary confirmatory serological test, indirect immunofluorescence (IIF, [28]). For direct molecular PRRSV detection, the Virotype PRRSV RT-PCR Kit (Qiagen, Hilden, Germany) was used according to the manufacturer’s instructions [29]. The sequences of the oligos of the kit are not commercially available.

### 2.3. Data Processing

The domestic pig and wild boar population densities were calculated by dividing the population size numbers with the given county’s surface area (km^2^). A linear regression model was then fitted to describe the relationship between the density of domestic pigs (*x*) and the density of wild boars (*y*) at the beginning of the PRRS eradication (2014), in the year of the ASF outbreak (2018), and after the end of the PRRS eradication (2023). The normality of the models’ residuals was tested using the Shapiro–Wilk test, and the dependence between the independent variables and the residuals were tested graphically. The explained variance rates (R^2^) and the parameter estimations were tested by two-tailed *t*-tests with a 95% CI. The analyses were performed in RStudio software with the “moments” package (2023.06.1+524) [30,31].

## 3. Results

**Figure 1:**
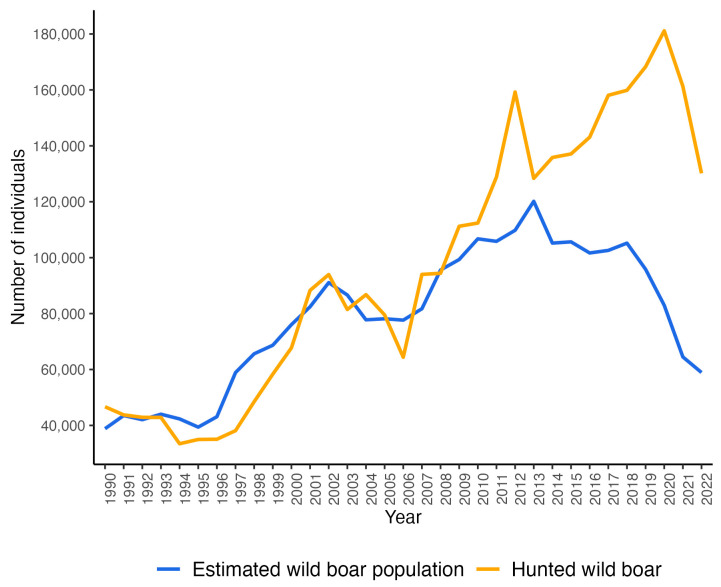
Estimated wild boar population and hunted wild boar individuals in Hungary between 1990 and 2022.

The annual numbers of the reported wild boar population in Hungary between 1990 and 2022 show an overall increasing trend until the last few years (Figure 1). The size of the population estimated in February each year exceeded 100,000 animals between 2010 and 2018, and then, due to the control measures introduced to prevent the spread of ASF (including population reduction), it decreased to below 60,000 by 2022. At the beginning of the PRRS eradication (2014), the wild boar population in Hungary reached its highest number in the last 33 years [26].

**Figure 2:**
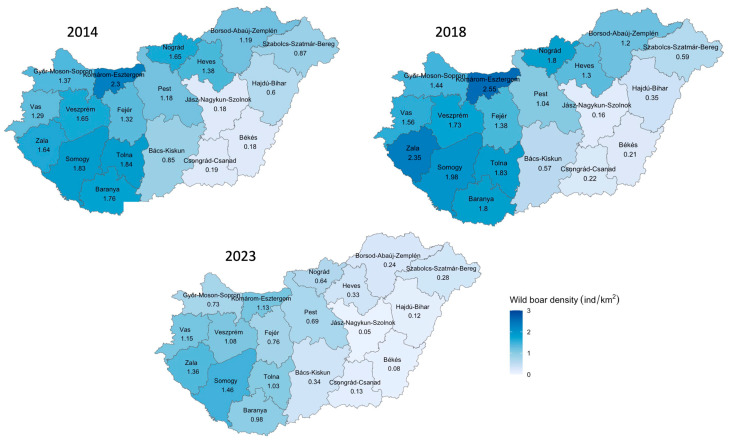
Density of the wild boar population (ind/km²) by county in Hungary in 2014, 2018, and 2023.

**Figure 3:**
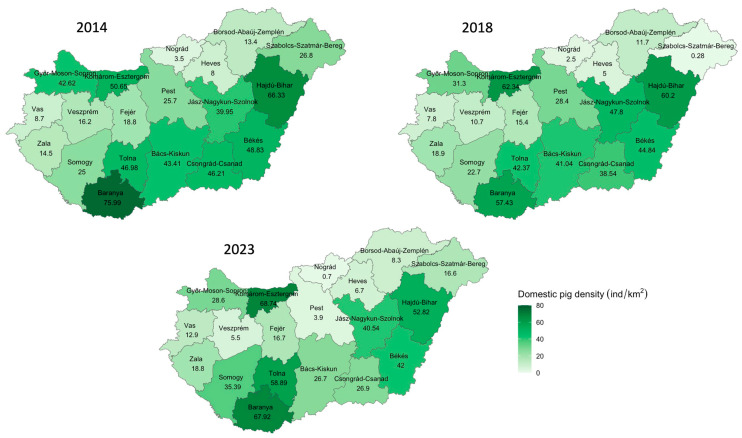
Density of the domestic pig population (ind/km²) by county in Hungary in 2014, 2018, and 2023.

The spatial distribution of the wild boar and domestic pig population densities in all 19 counties in the year of the beginning of the PRRS eradication (2014), in the year of the ASF outbreak (2018), and in the year after the end of the PRRS eradication (2023) shows an overall reduction in the wild boar density and little change in the domestic pig density (Figure 2 and Figure 3). While in 2014 and 2018, the wild boar density per km^2^ was 1.13 at the national level, this number decreased to 0.6 wild boar/km^2^ by 2023 in the context of the National Action Plan requirements published in 2021 [32].

**Figure 4:**
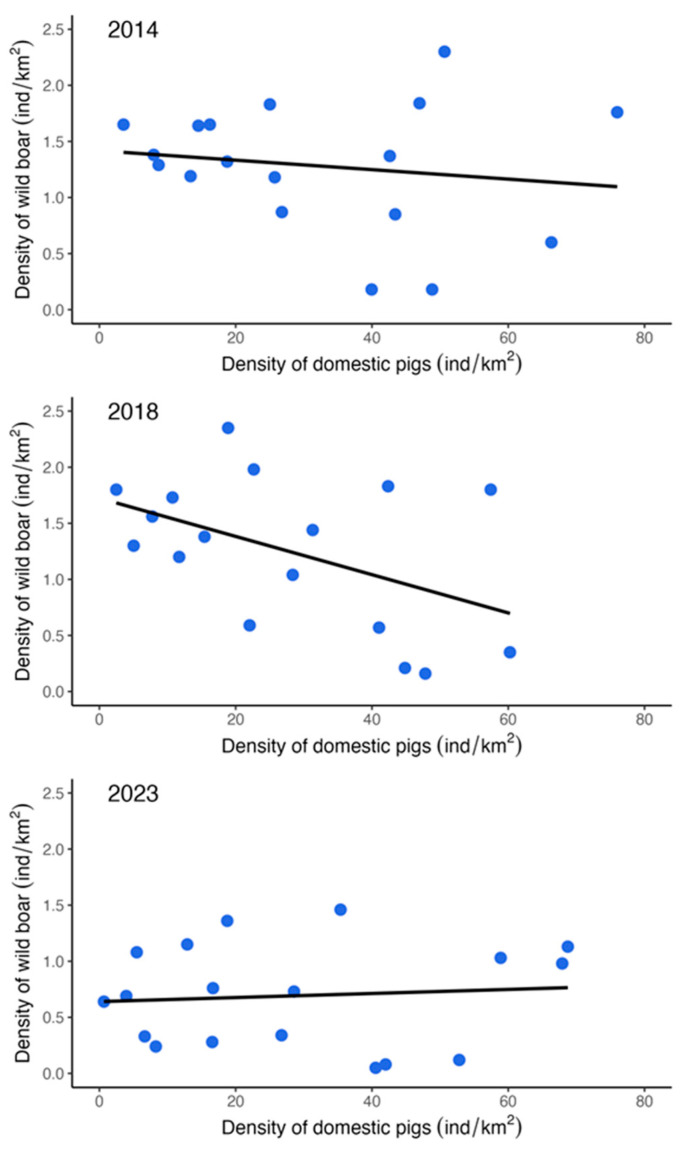
Relationship between domestic pig and wild boar density by county in Hungary in 2014, 2018, and 2023.

There was no relationship between the density of the wild boar and domestic pig populations by county in any of the three periods (Figure 4). For 2014, the linear model explains a statistically not significant and weak proportion of variance (R^2^ = 0.02, F(1, 16) = 0.39, *p* = 0.542). Therefore, the effect of domestic pig density is statistically non-significant and negative (beta = −4.22 × 10^−3^, 95% CI [−0.02, 0.01], t(16) = −0.62, *p* = 0.542; Std. beta = −0.15, 95% CI [−0.68, 0.37]). In 2018, a similar result can be seen (R^2^ = 0.04, F(1, 16) = 0.73, *p* = 0.404), also with a statistically non-significant and negative effect (beta = −7.62 × 10^−3^, 95% CI [−0.03, 0.01], t(16) = −0.86, *p* = 0.404; Std. beta = −0.21, 95% CI [−0.73, 0.31]). Also in 2023, a statistically not significant and very weak proportion of variance (R^2^ = 7.85 × 10^−3^, F(1, 16) = 0.13, *p* = 0.727) can be seen in the model with a statistically non-significant but positive effect of the density of domestic pigs (beta = 1.81 × 10^−3^, 95% CI [−8.97 × 10^−3^, 0.01], t(16) = 0.36, *p* = 0.727; Std. beta = 0.09, 95% CI [−0.44, 0.62]). The differing spatial distributions of wild boar and domestic pigs primarily result from ecological and habitat conditions for wild boar and economic and management factors for domestic pigs. By 2014 and 2018, the ratios of domestic pigs to wild boar in Hungary were 29.81 and 27.3, respectively, which increased to 45.84 in 2023 following a notable decrease in wild boar numbers. Furthermore, as illustrated in Figure 4, the wild boar density was significantly reduced in 2023 (to around 1.5 animals/km^2^), yet seven counties still exceeded the target density of 0.5 animals/km^2^.

The results of the PRRS serological and virological tests performed in wild boar populations show that between 2011 and 2023 a total of 2842 samples were tested by PRRS ELISA, and 12 cases (0.42%) were found to be serologically positive (Table 1). This level of seropositivity is lower than the specificity value of 96.6% of the Ingenasa PRRS ELISA, but higher than the 99.9% specificity of the IDEXX PRRS ELISA. [33]. All blood samples were reported to have been taken from live wild boar within enclosed areas during herd culling activities. The positive cases were all juveniles; no sex data are available. Upon retesting with IIF tests, seven seropositive animals from one herd were culled. Further tests did not confirm PRRS-specific antibodies in the remaining cases. Additionally, between 2011 and 2023, all 274 PRRS PCR tests were negative.

## 4. Discussion

We investigated if the wild boar population in Hungary poses any risk of PRRS transmission to domestic pigs by looking at their seroconversion rates and the relationship between the spatial distribution of the two species. Various studies confirm our results that wild boars pose minimal risk of disease transmission. In a study conducted in Germany from 2004 to 2007, they explored the spatial, temporal, host, and virological aspects of PRRS virus infection in wild boar populations [12]. Through the PCR testing of 531 samples from 52 hunts, they discovered the presence of both US and European PRRS virus subtypes, with 15.9% of samples testing positive (14.2% for the US strain and 6.2% for the EU strain, indicating co-infection in some cases) [12]. Sequencing of the ORF-1 region of the virus determined 99.3% similarity to Lelystad virus and 97% similarity to the US strain of PRRSV (VR 2332) [12]. The prevalence of the disease was not correlated with either the age or body weight of the animals, and no correlation was found with either the wild boar or domestic pig spatial density [12]. Their study was the first comprehensive evidence of PRRS virus infection in wild boar in Hungary. They suggested there is only a very weak relationship between wild boar and domestic pig herds in terms of PRRS virus infection [12]. Moreover, in France, Albina et al. (2000) [13] tested 909 samples for PRRS antibodies between 1993 and 1995 and found 33 (3.63%) were positive. According to their results, wild boar may be reservoirs of infectious diseases, including PRRS, although the seropositivity rate detected does not indicate a serious threat to domestic pig herds [13].

In the United States of America, Pedersen et al. (2018) [15] found antibodies against PRRSV in 68 (1.2%) of 5506 wild boar samples from different parts of the country. Similarly to the previously mentioned studies, they also found that wild boar cannot be a significant source of infection for domestic pigs. Also in northern European countries, between 2011 and 2015, 1597 tissue and serum samples from 44 districts of 10 counties in Lithuania were analysed; PRRSV PCR assays were found to be positive in 18.66% of the samples where only the European type occurred [16]. No statistically significant differences in the spatial distribution of positive samples were found. Although positive cases occurred in all age groups, a significantly higher proportion of infections occurred in young adult and adult animals than in animals less than 12 months old [16]. ORF5 sequencing of PCR positive samples detected viruses of virus type 1, subtypes 3 and 4 [16]. It is considered that the 3 and 4 subtypes of PRRSV type 1 detected in wild boar pose a significant threat to the Lithuanian domestic pig population because only subtype 2 is present in these pigs [16]. In Slovakia, in 2012, two (1.55%) positive cases were found in the PRRS PCR testing of 129 wild boar tissue samples [18]. Sequencing of the PRRSV ORF7 segment showed that the European strain of PRRSV was 99.7% and 100% similar to the PRRSV Lelystad reference and Porcilis PRRS vaccine strains, respectively [18]. Although they could not clarify the origin of the virus detected in wild boar, they considered it very likely that it was introduced into wild boar from vaccinated domestic pigs [18]. Further results from Spain showed that the prevalence of PRRS in wild boar may be influenced, although not significantly, by the density of domestic pigs [17]. ELISA testing of serum PRRS from 294 wild boars and 80 Iberian pigs showed that seven (2.38%) wild boars and one (1.25%) Iberian pig were seropositive [17]. All samples tested negative by PRRS PCR. It is concluded that PRRSV has a limited transmission between free-roaming Iberian pigs and wild boar in mutual contact with them [17]. In this study, only 12 (0.42%) cases of seropositivity were found by the PRRSV ELISA testing of blood samples from 2842 wild boar over 12 years. All 274 PRRSV PCR tests were negative.

These results indicate that PRRSV infection in wild boar populations has not played, does not play, and is not expected to play a major role in the future of the infection of domestic pig herds, including periods before, during, and after disease eradication efforts, as well as during maintenance phases. Moreover, the low risk of infection may be confirmed by the fact that camera trap surveys in Hungarian pig farms indicate that the probability of wild boar–domestic pig encounters and the probability of ASF transmission are minimal in intensive farms and when appropriate protection measures are followed [31]. However, to mitigate ASF transmission risk, the population of wild boars in Hungary with confirmed positive cases has been significantly decreased (from 20,000 to 4700 [26]). Furthermore, efforts to eliminate ASF from Hungarian pig herds [25], specifically aiming to maintain wild boar density below 0.5 animals per km^2^ nationwide by 2025, further ensure that PRRSV transmission between wild boars and domestic pig herds is unlikely.

## 5. Conclusions

This study reveals a minimal prevalence of PRRS within Hungarian wild boar populations, indicating a low risk of transmission to domestic pig herds. This confirms our experience that the source of infection in large-scale pig farms infected with PRRS virus is most often due to the transport of live animals and carcasses and the failure to comply with external disease control measures on the farm [24]. Therefore, we have found no evidence to suggest that wild boars are a source of infection.

## Figures and Tables

**Table 1:** Total wild boar Ingezim PRRS 2.0 and IXEXX PRRS X3 Ab tests (PRRS ELISA) and PRRS PCR tests taken between 2011 and 2023 in all 19 counties in Hungary.

County	PRRS ELISA		PRRS PCR	
	Total	Positive	Total	Positive
Bács-Kiskun	383	2	5	0
Baranya	15	0	0	0
Békés	287	0	3	0
Borsod-Abaúj-Zemplén	22	0	0	0
Csongrád	450	7	1	0
Fejér	10	0	11	0
Győr-Moson-Sopron	11	0	0	0
Hajdú-Bihar	234	0	2	0
Heves	18	0	16	0
Jász-Nagykun-Szolnok	532	1	1	0
Komárom-Esztergom	4	0	0	0
Nógrád	92	0	95	0
Pest	310	0	123	0
Somogy	16	0	1	0
Szabolcs-Szatmár-Bereg	159	0	14	0
Tolna	218	2	1	0
Vas	0	0	0	0
Veszprém	7	0	1	0
Zala	74	0	0	0
Total	2842	12	274	0

## Data Availability

The original data presented in this study are openly available at the National Game Management Database at https://www.ksh.hu/docs/hun/xstadat/xstadat_hosszu/h_omf001c.html and the Hungarian Central Statistical Office at https://www.ksh.hu/docs/hun/xstadat/xstadat_hosszu/h_omf001c.html (accessed on 21 March 2024).
